# Malaria mosquito control in rice paddy farms using biolarvicide mixed with fertilizer in Tanzania: semi-field experiments

**DOI:** 10.1186/s12936-019-2861-4

**Published:** 2019-07-08

**Authors:** Humphrey D. Mazigo, Leonard E. G. Mboera, Susan F. Rumisha, Eliningaya J. Kweka

**Affiliations:** 1 Department of Medical Parasitology, School of Medicine, Catholic University of Health and Allied Sciences-Bugando, P.O. Box 1464, Mwanza, Tanzania; 2 SACIDS Foundation for One Health, Sokoine University of Agriculture, P.O. Box 3297, Chuo Kikuu, Morogoro, Tanzania; 3 National Institute for Medical Research, Headquarters, P.O. Box 9653, Dar Es Salaam, Tanzania; 4 Division of Livestock and Human Diseases Vector Control, Mosquito Section, Tropical Pesticides Research Institute, P.O. Box 3024, Arusha, Tanzania

**Keywords:** Biolarvicide, Fertilizer, Malaria, Mosquitoes, Larvae, Rice fields, Tanzania

## Abstract

**Background:**

The wide distribution of malaria mosquito breeding sites within tropical environments limits the mosquito larval source management efforts to control malaria. Rice farming contributes substantially in supporting malaria mosquito productivity in tropical countries. To overcome this challenge, this study was carried out to determine the effect of applying a mixture of biolarvicide and fertilizer on mosquito larvae density in rice farms under semi-field conditions in Tanzania.

**Methods:**

A semi-field experiment was designed to determine the timing of application of a biolarvicide, *Bacillus thuringiensis israelensis* (Bti) and fertilizer (di-ammonium phosphate-DAP or urea) and assess their effect on mosquito larvae density and rice grain outputs. The experiment had five blocks (4 treatment arms and one control arm) and each had four replicates. Treatment arms had different intervals of days between treatments for mixtures of fertilizer and biolarvicides. The dosages used were 10 g of Bti/16 M^2^ and 160 g of DAP/Urea/16 m^2^.

**Results:**

In overall, the intervention blocks (with biolarvicide) had lowest mean mosquito larvae abundance compared to control block (F = 22.42, *P *< 0.001). Similarly, the control arm maintained highest density of *Anopheles gambiae* sensu lato larvae compared to interventions blocks (F = 21.6, *P *< 0.001). The best determined timing for application of Bti was in 7 and in 10 days (F = 3.753, *P *< 0.001). There was neither significant different in mean rice grain harvest per ten panicle (F = 1.453, *P *= 0.27) nor mean difference in rice grain harvest (F = 1.479, *P *= 0.26) per intervention arms.

**Conclusion:**

The findings of this study have shown that application of a mixture of Bti and fertilizer have impact on both mosquito larvae density and maintaining yield rice harvest. Thus, application of a combination of biolarvicide and fertilizer can be an alternative approach in malaria mosquito intervention among rice farming communities of rural Tanzania.

## Background

The rapid decline of malaria prevalence and parasitaemia in sub-Saharan Africa and many other malaria endemic countries has been noted in the last decade, from a mortality of 2.0 million to 436,000 per year in 2018 [1–3]. This has been mainly associated with wide use of malaria vector control measures, such as long-lasting insecticidal nets (LLINs) and indoor residual spray (IRS) as well as prompt diagnosis and effective treatment using artemisinin-based combination therapy [3–6]. However, the observed decline is faced by challenges of resurgence of residual malaria transmission [7], which are mainly attributed to changes in malaria vector behaviours [8], such as avoidance of house entry, diversion from contact with indoor treated surfaces or nets and early exit from houses [8–10]. There is also development of drug resistant in malaria parasites [11] and insecticide resistance among malaria vectors [12, 13]. In response to these challenges, the World Health Organization (WHO) is recommending the development and use of complementary measures to further reduce and possibly eliminate malaria [14]. One of such complementary control measure is the larval source management (LSM) using biolarvicides, which are considered to be relatively safe to human health and the environment [15, 16].

*Bacillus thuringiensis* var *israelensis* (*Bti*) is one of the recommended biolarvicides [17] and has been proved to be effective for field control of mosquito larvae [18]. Different formulation of this biolarvicide have been developed and tested in laboratory, semi-fields conditions and fields condition at variable ecological settings [18]. The *Bacillus thuringiensis* var *israelensis* biolarvicides based on Bti H-14 serotype, is a water dispersible granular formulation evaluated by the World Health Organization Pesticides Evaluation Scheme (WHOPES) and has been found to be effective for 2–7 days for malaria vectors larvae control in open water bodies and for container breeding mosquitoes (*Aedes aegypti* and *Aedes albopictus*) for 4–9 weeks [18]. *Bti* has been demonstrated to be safe to human, wildlife and other nontargeted organisms [18]. In general the efficacy of *Bti* preparation against malaria vectors larvae depends on the formulation suited to the biology and habitat of the targeted mosquito species [18]. Field studies in Tanzania have demonstrated the effects of biolarvicides on malaria vectors larvae density [19], malaria prevalence [20, 21] and have proved to be the most cost-effective mosquito interventions in rural setting [22].

Although the WHO emphasizes on integrated vector management (IVM) [23], targeting both immature and adult mosquitoes, the utilization of LSM have received little attention in malaria endemic countries [18]. Most of National Malaria Control Programmes (NMCP) in African countries have not implemented this approach. The available reports indicate that 48 malaria-endemic countries worldwide use larval control interventions in only defined foci for malaria transmission, of which only 18 countries are in sub-Saharan Africa [5]. A number of challenges that hinders the adoption of this approach by most of the NMCP in sub-Saharan Africa have been described elsewhere [24, 25]. The need for intensive labour forces to reach multiple malaria vectors breeding sites, especially in rural area, high operational costs of this intervention, poor knowledge on methods of implementing and monitoring the intervention, the short residual effect of biolarvicides formulations and small number of public health professional, present significant challenges in implementing this control approach [24, 25]. To overcome some of these challenges, especially the need for labour-intensive activities to reach multiple malaria breeding sites, integration of biolarvicides (*Bti*) and fertilizer application was recommended to rice farmers so that both fertilizer and biolarvicides can be concurrently applied to paddy fields, which coincidentally constitute the largest proportion of malaria vectors breeding sites. The objective of this study was to determine the effect of applying a mixture of biolarvicide and fertilizer on mosquito larvae density and rice grain outputs under semi-field conditions in a rural area of central Tanzania.

## Methods

### Study area

The study was carried out in Kilosa District (5°55′–7°53′ S; 36°30′–37°30 E) in southern part of central Tanzania. Kilangali Rice Seed Farm was selected for the experiments. The farm has a total area of 1200 ha which is used for rice production. The area is characterized by swampy flatland and wetlands lying on the Kilangali alluvial basin. The farm is bordered by the Kilangali village (58′0″ South, 37°5′0″ East) which is occupied by approximately 3500 inhabitants. Most of the communities in the area are involved in small-scale rice farming. The rice farms are rain fed or flooded using water from rivers/streams/canals. The most commonly grown rice variety is Saro5 (TXD 306), which is mainly used for lowland plantation. The timeframe from planting to harvesting of this variety of rice takes 100 to 140 days. Malaria is endemic in the area; baseline assessment of malaria prevalence before implementation of the study revealed a prevalence of 14.2% and 17.5% based on microscopic examination and rapid diagnostic test, respectively [26].

### Semi-field experimental design

The semi-field experiment had five blocks and each of the blocks had four replicates, making a total of 20 experimental plots (Table 1). Treatments and controls arms were randomly assigned to the blocks. Of the five blocks, four blocks constituted the intervention arm making a total of 16 intervention plots (Table 1). The size of each experimental plot was 4 × 4 m and the spacing between experimental arms were 1.5 m. The experimental blocks were separated by a 1.5 m (buffer zone) that was made by soil contour and filled with water.

**Table 1:**
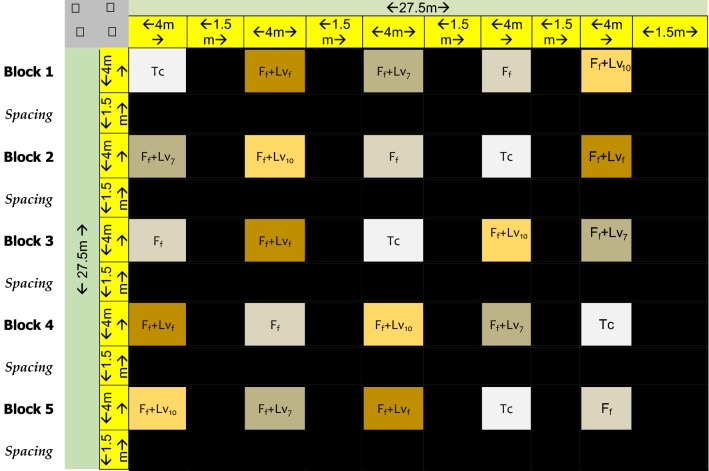
A randomized block design for allocations of replicates for each experimental (intervention) block and the control block

Tc: control, i.e. no fertilizer and no biolarvicides

F_f_: fertilizer following farmer’s schedule, i.e. applied on day 0, day 28 and day 60

F_f_ + Lv_7_: fertilizer following farmers schedule + biolarvicides applied every 7 days

F_f_ + Lv_10_: fertilizer following farmers schedule + biolarvicides applied every 10 days

F_f_ + Lv_f_: fertilizer + biolarvicides both following farmers schedule

In the experimental site, (Table 2), Block 1, was the control arm mimicking the practices of rice farming without fertilizer and no biolarvicide. Block 2 was an intervention in which fertilizer only [di-ammonium phosphate (DAP) for basal and Urea for top dressing] without biolarvicide was applied on day 0, day 28 and day 60 (Table 2). This mimicked the rice farmer’s fertilizer application schedules. Block 3 was an intervention arm, with an objective of mimicking the ideal application timing of fertilizer and biolarvicide in rice farms. Biolarvicide and DAP fertilizer was applied on day 0, followed by application of biolarvicide alone at every 7 days. On day 28, a mixture of biolarvicide and urea fertilizer was applied again. This was followed by application of biolarvicide only at every 7 days. At day 60, a mixture of biolarvicides and urea was applied again; this was followed by application of biolarvicide only at every 7 days until day 120, when no more water was put into the farm (Table 2). Block 4 was meant to mimic fertilizer application i.e. fertilizer timing (day 0, day 28 and day 60) but alter biolarvicide application to 10 days interval. In this block, a mixture of biolarvicide and DAP was applied on day 0, this was followed by application of biolarvicide only after every 10 days. On day 28, a mixture of biolarvicide and urea fertilizer was applied. This was then followed by application of biolarvicide only after every 10 days up to day 59. On day 60, a mixture of biolarvicide and urea was applied; this was followed by application of biolarvicide only after every 10 days until day 140 (harvesting time) (Table 2). Block 5 was an intervention arm, in which mixture of biolarvicide and fertilizer (DAP or Urea) was applied to mimic ideal fertilizer application timing during rice farming. A mixture of biolarvicide and DAP fertilizer was applied on day 0, this was followed by application of a mixture of biolarvicide and urea on day 28 and day 60 (Table 2).

**Table 2:**
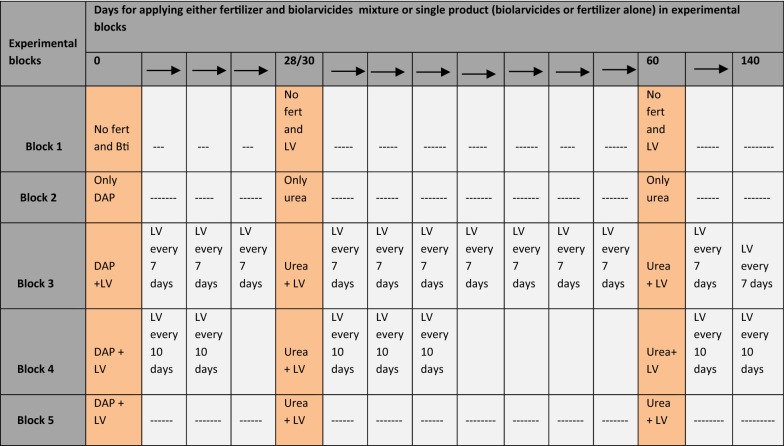
The application timing for biolarvicides (LV) and fertilizer (Urea/DAP) in each of the intervention arm

*DAP* di-ammonium phosphate, *LV* biolarvicides

### Application of biolarvicides and fertilizer

Biolarvicide, *Bacillus thuringiensis* var *israelensis*, strain AM-65-52) (VectoBac, Lot no. 251-997-N8, Valent Biosciences Corporation, USA) in form of granules and DAP or urea fertilizers in form of granules were applied either as single or mixed product. The dosages used were 10 g of Bti/16 M^2^ and 160 g of DAP/Urea/16 m^2^. Dosage calculation of *Bti* was based on the results of field studies in Tanzania [19] and fertilizer was based on the standard (40 kg per acre) provided by the Kilangali Rice Seed Farm management. Before application, biolarvicide and fertilizer were hand mixed thoroughly in order to get a homogenous mixture. Then, the mixture was applied by hand.

### Monitoring of mosquito larvae density

Mosquito larval density was monitored every day across the control and intervention blocks for a period of 140 days. Larvae densities were determined before the application of either biolarvicide or a mixed product of (biolarvicide and fertilizer) during each application day. To determine larval population density, a standard dipping technique described elsewhere was used [27]. Six dips were taken from each experimental plot to determine the larval density (counting number of larvae collected per dip). The collected larvae were all identified to species levels using the morphological identification keys [28]. The numbers of mosquito larvae counted were documented by species. Two field experienced entomology technicians participated in collecting and identifying collected mosquito larvae.

### Measuring the weight of rice grains per panicle and per treatment block

From each of the replicates throughout the intervention and control blocks, 10 panicles were selected at random by agricultural technicians. The grains in the panicles were separated manually and weighed using an electronic balance having a sensitivity of 0.1 g. The total harvest of rice grains from each replicate was weighed in kilograms and used to obtain the total rice grain harvest in each of the respective block [29].

### Data management and analysis

Data were entered into Microsoft Excel sheets, cleaned and analysed using IBM Statistical Package for Social Science (SPSS), version 25 (IBM Corp., Armonk, NY, USA). The general linear model univariate analysis was used for the comparison of larvae abundance by species in different treatment arms using larvae density as dependent variable, intervention as fixed factor and species composition as random factor. The means of mosquito larvae abundance were compared between the different species and treatment using the Turkey’s HSD-test. ANOVA was used to compare the mean weight of rice grain per 10 panicles and rice grain output measured in kilogram between interventions using the Tukey–Kramer test to separate the significance levels between the means [29].

## Results

### Mosquito larvae abundance by intervention arms

**Fig. 1:**
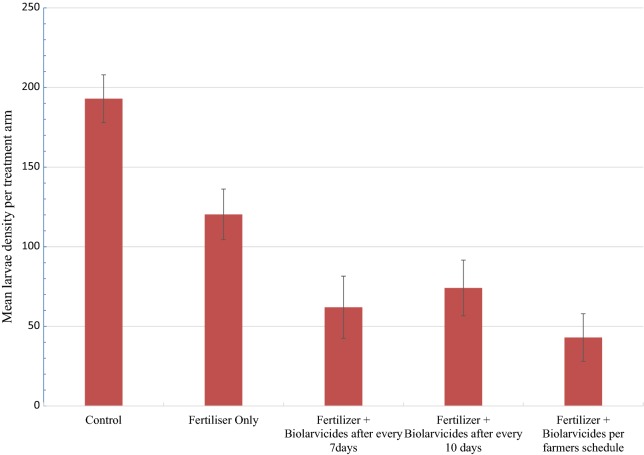
The mean overall mosquito larval abundance per intervention arm

**Fig. 2:**
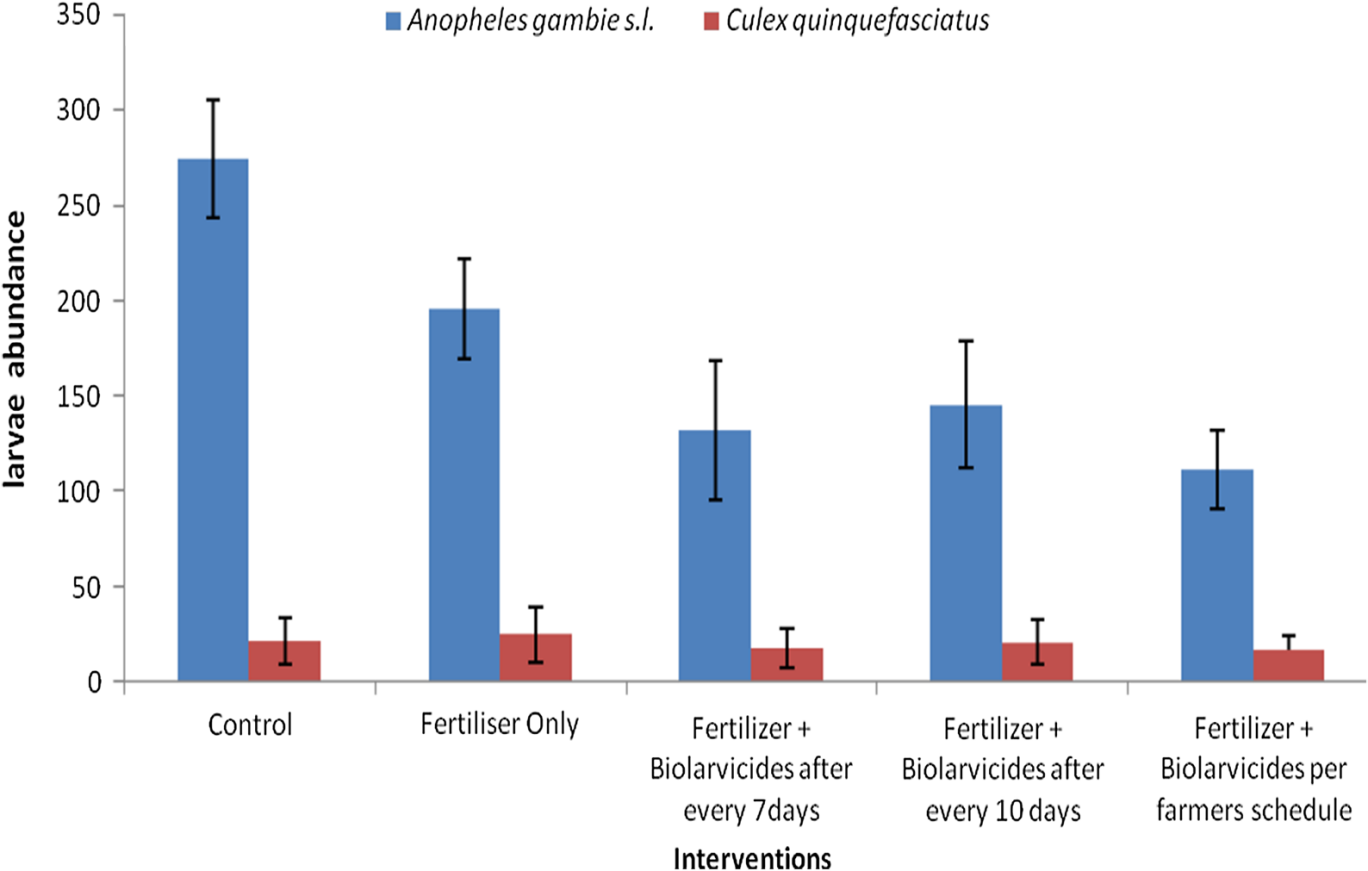
The mean *Anopheles gambiae* s.l. and *Culex quinquefasciatus* larvae abundance by intervention arms

Comparison of means of larvae abundance per intervention revealed that there was a statistically significant difference in mosquito larvae density between interventions (df = 4, F = 21.70, *P *< 0.001) (Figs. 1 and 2). The control block which received neither fertilizer nor biolarvicide had the highest mosquito larvae abundance.

### Mosquito larvae species composition and abundance

The mosquitoes larvae species composition identified were composed of *Anopheles gambiae* sensu lato (s.l.) and *Culex quinquefasciatus*. There was a statistically significant difference in abundance of *An. gambiae* s.l. between treatment blocks (df = 4, F = 21.6, *P *< 0.001), with the control block having the highest larvae density. However, there was no difference in density of *Culex quinquefasciatus* between treatment blocks (df = 4, F = 1.18, P = 0.12). Overall, there was a statistically significant difference in abundance of mosquito larvae between *An. gambiae* s.l. and *Culex quinquefasciatus* in each treatment blocks (Fig. 2).

### Comparison of mosquito larvae density

**Fig. 3:**
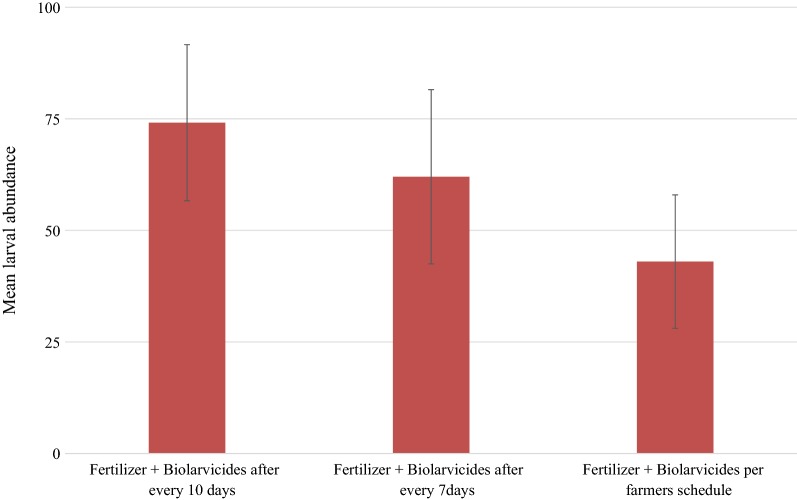
Mosquito larvae abundance for three intervention by time of application of biolarvicides and fertilizer mixture

To determine the best timing for application of biolarvicide and fertilizer which could have effects on the yield of paddy plants and mosquito larvae density, an overall comparison of mean mosquito larvae density by species for intervention arm 3, 4 and 5 was done. Overall, there was a statistical significance difference in mean mosquito larvae abundance (F = 3.753, *P *< 0.02), with intervention arm 5 having the highest mosquito larvae density (Fig. 3). No difference was observed between arms 3 and arm 4.

In relation to mosquito species abundances, overall, there was no mean difference in *An. gambiae* s.l. larvae abundance between the three intervention arms (F = 2.7, *P *= 0.07) (Fig. 3). However, the arm with fertilizer and biolarvicides following farmer’s schedule (arm 5) had the highest *An. gambiae* s.l. larvae abundance as compared to arm 3 and 4. A noted mean difference in *Culex quinquefasciatus* larvae abundance was observed (F = 4.6, *P *< 0.01) (Fig. 3).

### Comparison of mean weight per ten panicles and total rice grain harvest per treatment blocks

**Fig. 4:**
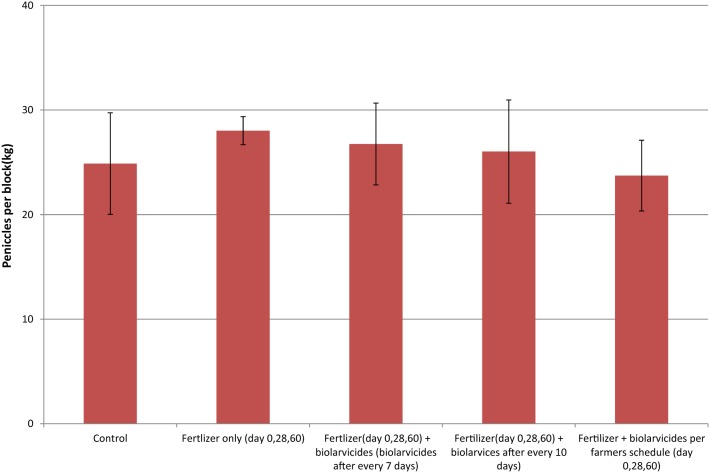
Mean harvest per panicles between and within the study arms

**Fig. 5:**
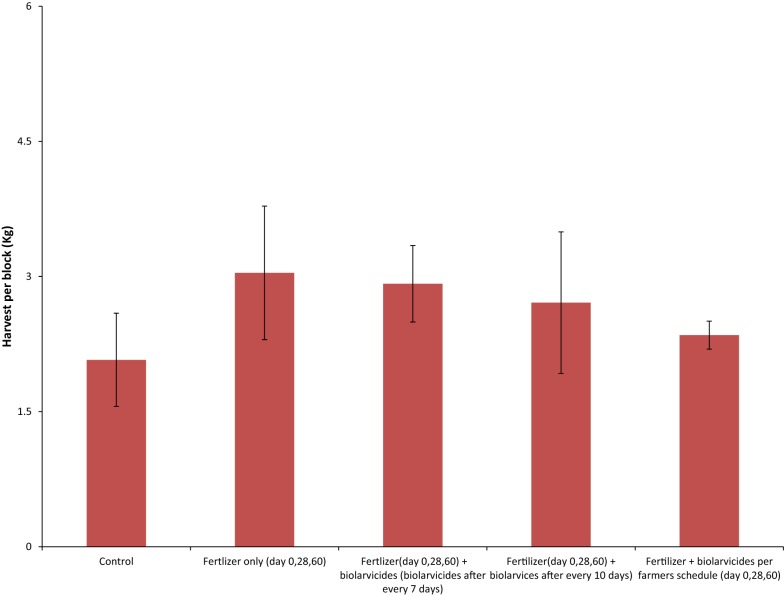
Mean rice grain harvest between intervention blocks

The mean weight of rice grain harvest per ten panicle varied from 23.7 g in arm 5 to 28.02 g in the arm 2. The comparison of mean rice grain harvest per ten panicles between the study arms was not statistically significant (F = 1.453, P = 0.27) (Fig. 4). The mean harvest per study arm varied from 2.1 kg in the control arm to 3.04 kg in arm 2 of the field experiment. The mean difference in rice grain harvest between the experimental arms was not statically significant (F = 1.479, *P *= 0.26) (Fig. 5). However, the control arm had the lowest mean harvest.

## Discussion

To achieve malaria elimination goals, the current malaria control approaches needs to be complemented with intervention measures which target the mosquito aquatic stages. The findings indicate that, in general application of biolarvicides as a single product or as a mixture of biolarvicides and fertilizer (DAP/urea) resulted into decline in mosquito larvae density. In comparison to intervention arms, the control arm had the highest mosquito larvae density. These findings noted a significant difference in *An. gambiae* s.l. larvae abundance between treatment arms with the control arm having the highest abundance. The treatment arm 3, 4 and 5 had the lowest *An. gambiae* s.l. larvae abundance. This study further assessed the best timing for application of biolarvicide either as a single product or combined with fertilizer and its effects on mosquito larvae density. These findings indicated that application of biolarvicide either as a single product or mixed with fertilizer at every 7 or 10 days had significant impact in reducing mosquito larvae density compared to the arm where biolarvicide and fertilizer were applied following rice farmers schedule for applying fertilizer. These findings indicate further that application of biolarvicide and fertilizer combination had no effect on rice grain harvest.

The findings on the reduction of mosquito larvae density following application of biolarvicides as a single product or mixed with fertilizer on mosquito larvae density are corroborated by previous studies elsewhere in Africa [17, 30]. In Tanzania, in rice field application of *Bti* only or in combination with *Bacillus sphaericus* have shown to provide more than 80% reduction of later instars of *Anopheles* and *Culex* species [15, 19]. In Western Kenya, application of *Bti* and *Bacillus sphaericus* biolarvicides in aquatic habitats reduced the proportion of aquatic habitats containing Anopheles larvae from 51% during the no-intervention periods to 7% during the intervention [15, 30]. The wide distribution of malaria vectors breeding sites in the tropical areas which are created by human activities such as in rice farming present a significant challenge to achieve maximum application of biolarvicides to these areas. To control mosquito density and reduce malaria transmission in rice farming agro-ecosystem, our innovation of mixing biolarvicides with fertilizers and use the farmer’s fertilizer application skills to reach multiple breeding sites in rice farms offers an opportunity to expand this intervention. This field experimental results clearly show that the efficacy of *Bti* on mosquito larvae stage is not affected by fertilizer. Thus, it is possible to incorporate the *Bti* granules in fertilizer bags at manufacturing stage and distribute a mixed product to rice farmers to apply it in rice fields during farming to control malaria vector density. This in turn, will have impact on incidence of clinical malaria and malaria vectors abundance [15, 17].

The findings on the best timing for applying biolarvicides either as a single product or in combination with fertilizer revealed that there was no mean difference in mosquito larvae density, especially *An. gambiae* s.l. larvae when biolarvicide was applied after every 7 or 10 days. However, when a mixture of biolarvicide and fertilizer was applied following the rice farmer’s schedule (at day 0, 28 and 60), the intervention arm was heavily re-populated by mosquito larvae 7–10 days post-application. This indicates that, the *Bti* remained only effective for 7–10 days. Previous studies in East Africa have shown some variation of the effective time period (residual effect) for *Bti* in field conditions. In Tanzania, field experiments in rice fields have reported that *Bti* remained effective for up to 14 days [15, 19]. In coastal areas of Kenya [31, 32] and in field experiments in India *Bti* was reported to remain effective for 2–9 days [33]. Cumulatively, current study results and those of others agree that, the best timing for biolarvicides either as a single product or a mixed product with fertilizer on average is between 7 and 10 days. The low residual effect of *Bti* raises the need for re-applying *Bti* after every 7–10 days [17, 30], which present a significant challenge to rice farmers and may affect the performance of the intervention. The invention of the long-lasting biolarvicides formulation that combines *Bti* and *Bacillus sphaericus* with potential for sustained release of the active ingredients for up to 6 months [33, 34], present a significant opportunity for the current innovation to be improved and make it more friendly and cost-effective, without the need for rice farmers to re-apply. However, this observation needs to be investigated.

On the other hand, the application of the mixture of *Bti* and fertilizer, did not affects the health of paddy plants and productivity of rice grains. These findings on the effects of fertilizer application as measured in terms of mean rice grain harvest per 10 panicles or mean weight per intervention arm was comparable to findings of similar studies in India [29] and Iran [35]. These indicate that, *Bti* mixed in fertilizer did not affect the efficacy of the fertilizer on plant health and rice grain productivity. On the other hand, the findings in Kenya that *Anopheles arabiensis* and *Culex quinquefasciatus* have preference to oviposit in fertilizer treated areas [36] is likely to be useful in the approach of using biolarvicide and fertilizer mixture in the control of mosquitoes. The fertilizer is likely to attract mosquitoes to lay their eggs in biolarvide treated areas, and hence maximize its killing effect.

## Conclusion

The findings of this study suggest that, application of *Bti* as single product or as mixture of *Bti* and fertilizer at an interval of 7–10 days reduce mosquito larvae density in rice fields. Using this innovation, *Bti* can be applied in a large area at a very low costs and this in turn will have impact on malaria prevalence while improving rice grain output. Further studies are recommended on the following areas (i) integrate the slow releasing long acting biolarvicides into rice farmer’s fertilizer application skills and assess its effects on malaria transmission indices, (ii) the fact that *Bti* and fertilizer have different pH range, it is important to understand if the pH of the fertilizer affects the effectiveness of the *Bti* when the two product are mixed together and stored for long time and (iii) assesses the impact of the innovation in areas with different malaria transmission levels or in areas with wide coverage of other malaria intervention measures, to assess its contribution in reducing clinical malaria, malaria vectors larvae abundance and indoor adult density.

## Data Availability

All relevant data supporting the conclusion of this article are included within the article.

## Abbreviations

DAP: di-ammonium phosphate
Bti: *Bacillus thuringiensis* var *israelensis*
IVM: integrated vector management
LLINs: long-lasting insecticidal nets
LSM: larval source management
NMCP: National Malaria Control Programme
WHO: World Health Organization
